# The Relationship between Paresthesia and the Presence of Cardiac Dysautonomia in Patients with Post-COVID-19 Syndrome: A Preliminary Observational Study

**DOI:** 10.3390/brainsci13071095

**Published:** 2023-07-20

**Authors:** Erislandis López-Galán, Arquímedes Montoya-Pedrón, Miguel Enrique Sánchez-Hechavarría, Mario Eugenio Muñoz-Bustos, Gustavo Alejandro Muñoz-Bustos

**Affiliations:** 1 Facultad de Medicina 2, Universidad de Ciencias Médicas de Santiago de Cuba, Santiago de Cuba 90100, Cuba; erislandislopez@infomed.sld.cu; 2 Departamento de Neurofisiología Clínica, Hospital Clínico Quirúrgico Juan Brunos Zayas Alfonso, Universidad de Ciencias Médicas de Santiago de Cuba, Santiago de Cuba 90100, Cuba; arqui6606@gmail.com; 3 Departamento de Ciencias Clínicas y Preclínicas, Facultad de Medicina, Universidad Católica de la Santísima Concepción, Concepción 4090541, Chile; 4 Núcleo Científico de Ciencias de la Salud, Facultad de Ciencias de la Salud, Universidad Adventista de Chile, Chillán 3780000, Chile; 5 Departamento de Kinesiología, Facultad de Medicina, Universidad de Concepción, Concepción 4030000, Chile; marmunozb@udec.cl; 6 Escuela de Kinesiología, Facultad de Salud y Ciencias Sociales, Campus El Boldal, Sede Concepción, Universidad de Las Américas, Concepción 4030000, Chile

**Keywords:** COVID-19, post-COVID-19 syndrome, autonomic nervous system, dysautonomia, autonomic function test, heart rate variability, paresthesia

## Abstract

Introduction: Post-Coronavirus disease 2019 (Post-COVID-19) syndrome has neurological symptoms related to the dysfunction of the autonomous nerve system. However, a pathogenic relationship between post-COVID-19 syndrome and dysautonomia still remains to be demonstrated. Establishing a pathogenic relationship between paresthesia and the presence of cardiac dysautonomia in patients with post-COVID-19 syndrome is the objective of this study. Participants and Methods: This observational study was carried out in the neurophysiology service wing of the Juan Bruno Zayas Hospital, Santiago de Cuba, in Cuba. The patients were recruited through a post-COVID-19 clinic at the same hospital. A variability study of cardiac frequency and a test of autonomic cardiovascular reflexes was carried out, which is composed of deep breathing, orthostatism, and the Valsalva maneuver. Results: The variability parameters of the cardiac frequency, the expiration–inspiration ratio between deep breaths, and the Valsalva Index showed no statistically significant differences between healthy participants and those with post-COVID-19 syndrome. During the Valsalva maneuver, there was a greater cardiac frequency response in participants with post-COVID-19 syndrome than in healthy subjects. The difference in supine and standing blood pressure was significantly minor in patients with post-COVID-19 syndrome. The logarithm of high frequency (log HF) increased significantly in patients with paresthesia when compared to patients without paresthesia. Conclusions: In the autonomic function tests, no signs of dysautonomia were found in patients with post-COVID-19 syndrome. The presence of paresthesias is associated with differences in cardiac vagal activity, which may suggest that damage to peripheral sensory nerve fibers could be associated with an affectation to autonomic fibres.

## 1. Introduction

The severe acute respiratory syndrome Coronavirus 2 (SARS-CoV-2) is the pathogenic agent of the Coronavirus disease 2019 (COVID-19) that has spread throughout the world. Although respiratory alterations are more common, there is an increasing amount of evidence of the neurological damage caused by SARS-CoV-2, estimated to have affected at least a third of COVID-19 patients [1,2]. The presence of viral particulates has been found in the cerebral tissue of deceased patients, and the presence of SARS-CoV-2 has been found in the cerebrospinal fluid of a COVID-19 patient [3]. Moreover, it has been stated that SARS-CoV-2 can enter the central nervous system through various mechanisms, such as invading the olfactory epithelium, the synapses of nerve endings, and the blood–brain barrier by endothelial damage [4].

Patients that have recovered from COVID-19 can present a wider variety of symptoms that, when they persist for over 12 weeks, are defined as “post-COVID-19 syndrome” or “long COVID-19” [5,6]. The careful diagnostic evaluation generally is unable to identify the specific causes of post-COVID-19 syndrome, although some post-COVID-19 symptoms, including those of cardiovascular origin, could be related to anomalies of the autonomic nervous system (ANS) [5,7]. Because of this, and keeping in mind the possible role of the ANS on COVID-19 symptoms, there has been a proposal for monitoring the vagal tone as a predictive marker for the course of this illness [7].

The ANS is a complex network originating from the brain, the brain stem, the spinal cord, the heart, and extracardiac organs [8] that play an important role in regulating the homeostasis of the entire body [7]. Symptoms of dysautonomia in patients with COVID-19 have been observed, which include diarrhea and sweat disorders [9]. The physiopathological mechanisms responsible for the deterioration of the ANS are still speculated and could include direct viral damage, toxic effects from cytokines, and immune responses produced by viral components [5,10]. Regardless of the mechanism, the possible participation of the ANS in the SARS-CoV-2 infection is backed by the frequent emergence of neurological symptoms (anosmia and dysgeusia) [5], as well as the sporadic emergence of conditions typically related to the dysfunction of the ANS (orthostatic hypotension and orthostatic tachycardia) in post-COVID-19 syndrome [5,6,10,11].

The evaluation of the ANS can be obtained from a combination of microneurography and continuous measurements of catecholamines; however, such measurements are difficult to obtain. On the other hand, it has been shown that autonomic activity can be evaluated through the specific analysis of heart rate variability (HRV) [2,12]. However, the studies on COVID-19 are not conclusive and present contradictory results. It has been reported that COVID-19 patients who have recovered, in comparison to healthy controls, present reduced HRV parameters [5,10,13] and that in patients with severe conditions, it is also lower than those with lesser or asymptomatic conditions [7]. Another study demonstrated an increase in the parasympathetic activity of patients with COVID-19, independently of confusion factors such as age, sex, and comorbidity [10]. HRV analysis parameters in the time domain, such as the square root of the mean squared differences of successive RR intervals (RMSSD) and the standard deviation of the RR intervals (SDNN), were significantly higher in patients with a history of COVID-19 and revealed parasympathetic hypertonia. All of this suggests that orthostatic symptoms that occur after COVID-19 could be associated with an autonomic imbalance [14].

On the other hand, the HRV in the time domain (SDNN, SDANN, NN50, and RMSSD) and in the frequency domain of low frequency (LF, 0.04–0.15 Hz) and high-frequency bands (HF, 0.15–0.4 Hz), did not show significant differences between the COVID+ and COVID− groups [1]. Another study used the Ewing battery of autonomic function tests, including deep breathing, active standing, and the Valsalva maneuver, to compare post-COVID-19 patients with and without fatigue. There were no significant differences between the groups, and no objective findings of autonomic dysfunction were observed [15]. However, this study did not compare post-COVID-19 patients with healthy controls.

Vagal neurostimulation attenuates inflammation both in experimental models and in preliminary human data, so it could have therapeutic benefits when combined with current medical strategies [16]. However, a pathogenic relationship between post-COVID-19 syndrome and dysautonomia still remains to be demonstrated [5]. Establishing a pathogenic relationship between paresthesia and the presence of cardiac dysautonomia in patients with post-COVID-19 syndrome is the objective of this study. This relationship would be important because it could help guide the management of this clinical condition.

## 2. Subjects and Methods

### 2.1. Place of Study and Participants 

This observational study was carried out in the neurophysiology service of the Juan Bruno Zayas Hospital in Santiago de Cuba, Cuba. Patients were recruited through the post-COVID-19 clinic at the same hospital between August and October 2021, and healthy subjects were obtained from people who volunteered for the study call. The inclusion criteria were patients who had a positive SARS-CoV-2 RT-PCR test at the time of the acute illness, who were older than 18 years, and with symptoms that did not present before the illness but that persisted beyond the 12 weeks after having tested negative for the SARS-CoV-2 RT-PCR. Patients with active COVID-19 infection, diabetes mellitus, use of beta-blockers or calcium antagonists, heart disease, left coronary artery disease, cardiac arrhythmias, chronic obstructive pulmonary disease, asthma, obstructive sleep apnea, BMI greater than 30 kg/m^2^, renal failure, cerebrovascular disease, thyroid disease, liver disease, and inflammatory and autoimmune disorders, or who were unable to complete any part of the assessment were excluded. The study was conducted in accordance with the Declaration of Helsinki, and the written informed consent of all participants was obtained. Approval for the present study was obtained from the Hospital’s Research Ethics Committee (ethical code: 51.2021). We conducted an a priori power analysis to check which sample size would be adequate for the statistical tests. For the power analysis, we used G*Power Version 3.1.9.6 software [17]. For sample size calculation, was employed a characterization of cardiac autonomic function in COVID-19 using heart rate variability [2] that reported a large effect (d = 1.01) (*p* = 0.001) in the comparison of the high frequency in healthy subjects and patients with COVID-19. Based on a power analysis of 0.95, a sample size of 44 participants would be sufficient to replicate these results.

### 2.2. Autonomic Tests

After two minutes of sitting, the patients rested in a supine position for ten minutes until fully relaxed. Resting blood pressure was then taken with a calibrated aneroid sphygmomanometer, and the ECG signal was registered for five minutes. The cardiovascular autonomic reflex test was then performed, consisting of deep breaths, orthostatism, and the Valsalva maneuver. Patients were asked to stand up as quickly as possible under the supervision of the physician and continued to stand for two minutes; they were asked to report any symptoms of light-headedness, dizziness, palpitations, or chest discomfort, and the blood pressure response to active standing. Subsequently, the patients were seated in a chair for two minutes and were instructed to perform the Valsalva maneuver by forced expiration at a pressure of 40 mmHg for a minimum of 15 s. The patients were then instructed to inhale and exhale deeply and under control at a rate of 6 breaths per minute.

A bipolar ECG was recorded in the MEDICID 5 equipment from Neuronic SA; the electrodes were placed according to the modified derivation II (negative electrode under the right clavicle and the positive one in the lower part of the left torso). Sampling frequency was 200 Hz, and the bandwidth hovered between 0.5–30 Hz. Data was extracted by an expert unaware of the patient’s diagnosis, and HRV analysis was performed using the VFC-32 software [18] from Universidad de la Habana.

### 2.3. Statistical Analysis

Statistical analysis was performed using the JASP software (version 0.16; https://jasp-stats.org (accessed on 31 January 2023)), with a statistical significance of *p* < 0.05. Descriptive statistics are reported as means and standard deviations (SD) or medians and interquartile range (IQR), as appropriate. A univariate analysis was performed with demographic variables and autonomic test results to examine the differences between COVID+ and COVID− individuals, using the *t*-test (t), the Mann–Whitney U (z) test, and the Chi-squared (χ^2^) test as appropriate after the Shapiro–Wilk normality test. The Octave software 2019 (version 5.1.0; https://www.gnu.org/software/octave/download.html (accessed on 15 January 2023)) was used to prepare the graphs of changes in heart rate (HR).

## 3. Results

**Figure 1:**
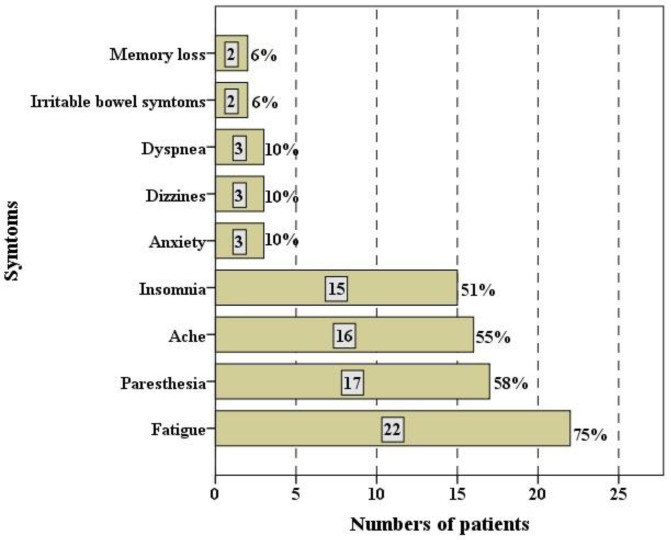
Persistent symptoms after COVID-19 infection.

In total, 44 participants were recruited for the study, which included 29 patients with long COVID-19 (onset time after recovery: 4.14 ± 1.74 months) and 15 healthy individuals who tested negative for SARS-CoV-2. The mean age of the long COVID-19 population was 45 (±12.1) years, with 6 (20.68%) male subjects and 23 (79.31%) female subjects. The most frequent symptoms found in this study were fatigue, with 22 subjects (75%), paresthesia, with 17 (58%), and pain, with 16 (55%) (Figure 1). Comparing patients with long COVID-19 and healthy subjects, both groups were matched for age and Body Mass Index (BMI), but not in gender. 

**Figure 2:**
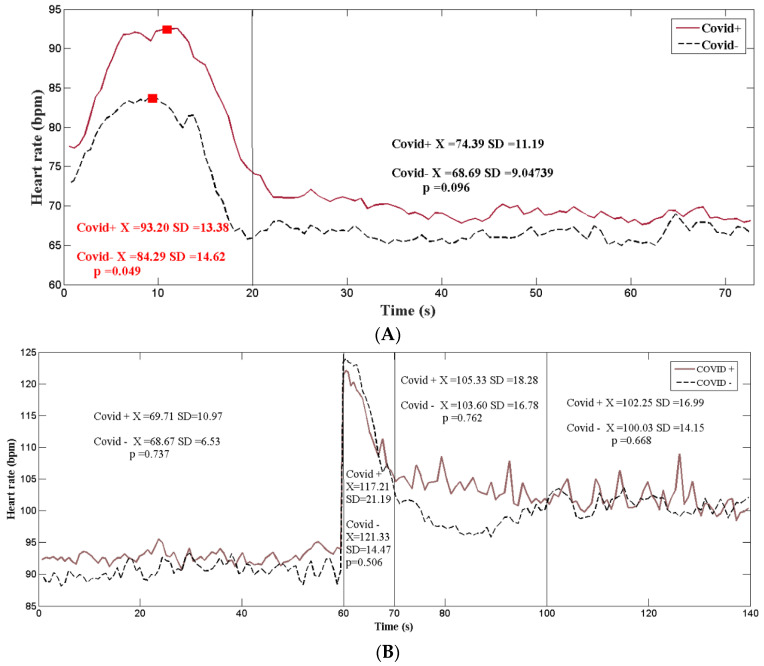
Changes in heart rate during the Valsalva maneuver (**A**) and during active standing (**B**).

**Figure 3:**
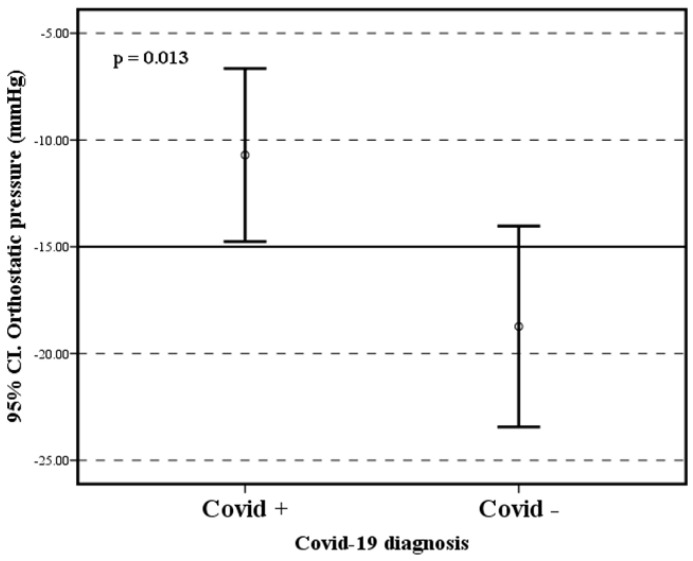
Differences between systolic blood pressure in the supine position and in response to active standing.

Parameters such as heart rate, systolic and diastolic blood pressure did not show statistically significant differences between the study groups. There were no differences between groups between time domain HRV parameters (RMSSD, SDNN, and CV) and frequency domain parameters (HF, LF, and LF/HF). All patients underwent autonomic testing according to the protocol. There were no statistically significant differences in global heart rate variability and expiration–inspiration ratio in response to the deep breathing test. The Valsalva maneuver did not demonstrate statistically significant differences in heart rate index, but there was a higher maximum heart rate in response to the Valsalva maneuver in subjects with long COVID-19 than in healthy subjects (Figure 2A). There were no statistically significant differences in heart rate variability before active standing (Figure 2B). After active standing, 20/29 (68.96%) of the patients in the cohort of long-term COVID-19 patients reported at least one of the following symptoms: palpitations, dizziness, and light-headedness. No subject in the cohort of healthy individuals reported any symptoms during active standing. There were no statistically significant differences in HR between the study groups at any time during active standing. There was a stabilization of the heart rate at 20 s after active standing in the group of healthy individuals, while in the prolonged COVID-19 group, it was at 40 s (Figure 2A). In addition, there were differences in systolic blood pressure in response to active standing between the two groups (Figure 3). The results of the statistical tests of these measures are shown in Table 1.

**Figure 4:**
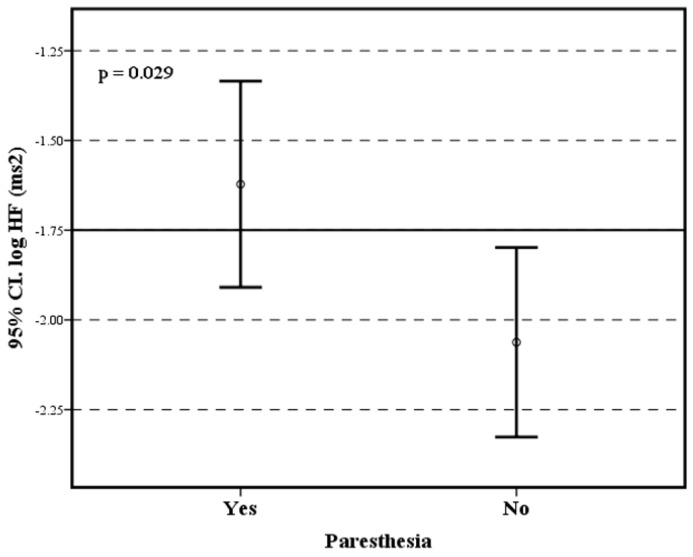
Differences in log HF between patients with post-COVID-19 syndrome with paresthesia and without paresthesia.

Among frequency-domain heart rate variability parameters, HF recording was significantly higher among long COVID-19 patients with paresthesia compared with patients without paresthesia (Figure 4). Bearing in mind that the patients without paresthesias were older, even so, the age-dependent heart rate variability parameters did not show significant differences between the two groups (Table 2). 

## 4. Discussion

In our study, fatigue was the most common symptom in patients with post-COVID-19 syndrome, followed by paresthesia and pain. In hospitalized Italian patients, 53% had fatigue, 43% had dyspnea, and 22% experienced chest pain after 2 months. After 4 to 8 weeks of recovery from COVID-19, fatigue is present in more than two-thirds of patients, followed by shortness of breath and symptoms of post-traumatic stress disorder [6]. Vital parameters such as HR, systolic (SBP), and diastolic (DBP) blood pressure did not show differences between the study groups; similar results were found in another study when comparing COVID-19 patients and healthy people [2].

SARS-CoV-2 is known to affect endothelial cells during acute infection, which can lead to autonomic dysfunction [15]. However, in light of current reports, the evidence for autonomic nervous system impairment caused by COVID-19 is not entirely clear. In the present study, the activity of the autonomic nervous system was evaluated in patients with post-COVID-19 syndrome in comparison with healthy subjects, showing that HRV parameters are not different between both groups. On their end, the cardiovascular autonomic reflex test did not show significant differences between the groups when the traditional parameters were analyzed, but when the dynamics of the ANS activation process during the Valsalva maneuver were considered, it was observed that patients with post-COVID-19 syndrome had a higher maximum HR than the healthy ones. In addition, patients with post-COVID-19 syndrome presented a smaller difference between systolic blood pressure lying down and standing up. However, there were no significant differences in HR during orthostatism between the two groups. All of this suggests a pattern of atypical autonomic damage in patients with post-COVID-19 syndrome, which makes it difficult to determine using conventional tests.

In this study, patients with paresthesia had higher parasympathetic tone than those without paresthesia, as demonstrated by the log HF parameter of HRV. Taking into account that the patients without paresthesias are older, even so, the age-dependent HRV parameters did not show significant differences between the two groups. It could be that these findings are coincidental since HF correlates closely with time domain parameters, which were found to be normal in this population. Another interpretation could be that the sensory damage caused by COVID-19 is also associated with an autonomic alteration. Paresthesia has been reported in patients with COVID-19 and is a symptom that evidences neurological damage [4]. When comparing patients with COVID-19 and healthy subjects, a significant decrease in HF and LF potencies was demonstrated in patients with COVID-19. The mean RMSSD was significantly higher in the COVID-19 group compared to healthy individuals, and the mean SDNN was higher among COVID-19 subjects trending toward statistical significance. These variables indicated that COVID-19 patients had higher parasympathetic tone than healthy subjects [2]. All this suggests that patients suffering from COVID-19 suffer from a state of irritation of the parasympathetic nerves, which causes their hyperactivity. However, this type of alteration is not a universal finding, so it could depend on the patient’s own genetic factors, as well as the SARS-CoV-2 variant. However, there is no published evidence to support these mechanisms.

In another study, they observed significant differences in HRV rate domain parameters between patients with severe and mild cardiovascular complications, possibly as signs of impaired ANS function [19]. On the other hand, it has been reported that sudden and severe confinement caused an increase in many subjective and objective stressors, in addition to drastically limiting the possibilities of physical activity. This likely resulted in a stressful situation that may lead to negative psychological and/or cardiovascular outcomes (i.e., decreased RMSSD and HF and increased resting heart rate) in the population [20].

The long-term prognosis of patients with post-COVID-19 syndrome in terms of cardiovascular effects and other sequelae is still unknown. Recent case reports and studies revealed that many patients developed postural orthostatic tachycardia syndrome (POTS) after COVID-19. These patients require longer visits and need more clinical resources for a comprehensive diagnostic evaluation. Although most people with COVID-19 make a full recovery, some patients continue to have chronic and diverse symptoms, including autonomic manifestations. Prolonged parasympathetic activity could be responsible for these symptoms [14]. Kurtoğlu E et al. [13] demonstrated that HRV was significantly depressed in patients after recovery from COVID-19, reflecting some degree of autonomic dysfunction in this group of patients. However, the decrease in HRV reflects autonomic dysfunction but does not distinguish between specific changes in sympathetic and parasympathetic activity. It seems clear that the type of autonomic alteration is parasympathetic hypertonia with sympathovagal imbalance associated with hypotension and orthostatic tachycardia described in these patients. Another important element to take into account in this study is the fact that it does not clarify the post-COVID-19 time. Further prospective and follow-up studies are needed to interpret the effect of parasympathetic dominance on prognosis in post-COVID-19 syndrome.

Our study has some limitations, mainly associated with the relatively low number of patients. Mild or severe symptomatic patients with SARS-CoV-2 infection were not divided. Factors such as stress, hypercytokinemia, prolonged hospitalization, and psychomorbidity, which typically characterize these patients, were not measured and could have a confounding effect. Despite these limitations, our results show that analyzing the classic HRV variables does not provide evidence of cardiovascular autonomic neuropathy in the prolonged phase of COVID-19. However, evidence is obtained of a sympathovagal imbalance with a predominance of parasympathetic activity. The strength of this study lies in suggesting the association between paresthesia and parasympathetic hypertonia in post-COVID-19 syndrome, which could at least partially explain the clinical manifestations in this stage of the disease, such as hypotension and orthostatic tachycardia.

## 5. Conclusions

In the autonomic function test, no signs of dysautonomia were found in patients with post-COVID-19 syndrome. The presence of paresthesias is associated with differences in cardiac vagal activity, which may suggest that damage to peripheral sensory nerve fibers could be associated with an affectation to autonomic fibers.

## Figures and Tables

**Table 1:** Demographic data and autonomic measurements.

Variables	COVID+ (*n* = 29)	COVID− (*n* = 15)	Statistic
Gender, female, *n* (%)	23 (79.31)	7 (46.66)	χ^2^ = 4.856 *p* = 0.028
	$\bar{x}$ (±SD)	$\bar{x}$ (±SD)	
Age	45.21 (±12.1)	44.80 (±8.7)	t = 0.693 *p* = 0.492
BMI	25.75 (±5.0)	25.94 (±3.5)	t = −0.123 *p* = 0.902
HR	70.14 (±10.9)	68.66 (±6.5)	t = 0.477 *p* = 0.635
SBP	120.77 (±17.6)	124.60(±22.3)	t = −0.610 *p* = 0.544
DBP	78.70 (±8.9)	82.46 (±15.5)	t = −0.999 *p* = 0.323
MBP	92.72 (10.73)	96.50 (17.50705)	t = −0.870 *p* = 0.389
Active stand SBP	131.48 (±18.9)	143.33 (±23.5)	t = −1.783 *p* = 0.082
Active stand DBP	90.74 (±13.7)	98.33 (±17.07)	t = −1.570 *p* = 0.124
Active stand MBP	104.31(±14.6)	113.28 (±18.5)	t = −1.725 *p* = 0.092
SDNN	45.96 (±27.4)	44.73 (±17.8)	t = 0.155 *p* = 0.877
CV	5.08 (±2.4)	5.01 (±1.7)	t = 0.104 *p* = 0.917
RMSSD	39.57 (±35.4)	37.55 (±24.4)	t = 0.195 *p* = 0.845
log LF	−1.756 (±0.51)	−1.535 (±0.35)	t = −1.476 *p* = 0.147
log HF	−1.801 (±0.52)	−1.602 (±0.36)	t = −1.302 *p* = 0.200
LF/HF	1.6554 (±1.439)	1.2572 (±0.811)	t = 0.984 *p* = 0.330
HRV (deep breathing)	26.851 (±11.9)	26.200 (±7.7)	t = 0.190 *p* = 0.850
EI	1.3155 (±0.1)	1.3466 (±0.1)	t = −0.687 *p* = 0.495
VI	1.6707 (±0.4)	1.5433 (±0.2)	t = 0.912 *p* = 0.366
Active stand HR	1.2646 (±0.5)	1.2673 (±0.1)	t = −0.020 *p* = 0.984
Active stand SBP	−10.703 (±10.2)	−18.733 (±8.4)	t = 6.655 *p* = 0.013

$\bar{x}$ = Mean, SD = Standard Deviation, BMI = Body Mass Index, HR = Heart Rate, SBP = Systolic Blood Pressure, DBP = Diastolic Blood Pressure, MAP = Mean Arterial Pressure, SDNN = Standard Deviation of RR intervals, CV = Coefficient of Variation, RMSSD = Square Root of mean value of the sum of the squared differences of the successive RR intervals, log LF = logarithm of low frequencies, log HF = logarithm of high frequencies, LF/HF = low frequencies/high frequencies, HRV (deep breathing)= Frequency variability heart rate during deep breaths, EI = expiration/inspiration ratio, IV = Valsalva index, ΔSBP = SBP change while standing.

**Table 2:** Comparison of the clinical profile, HRV parameters, and autonomic reflex test between patients with post-COVID-19 syndrome with and without paresthesia.

Variables	Paresthesia		Statistic
	Yes (*n* = 17)	No (*n* = 12)	
Gender, female, *n* (%)	14 (87.5)	9 (81.8)	χ^2^ = 2.32 *p* = 0.630
	$\bar{x}$ (±SD)		
Age	43.62 (±6.1)	51.90 (±13.4)	t = −2.169 *p* = 0.039
BMI	26.10 (±5.5)	25.25 (±4.4)	t = 0.423 *p* = 0.675
log LF	−1.64 (±0.4)	−1.91 (±0.6)	t = 1.361 *p* = 0.185
log HF	−1.62 (±0.5)	−2.06 (±0.3)	t = 2.314 *p* = 0.029
LF/HF	1.22 (±0.9)	2.28 (±1.8)	t = −1.978 *p* = 0.058
Entropy	−4.92 (±0.6)	−4.58 (±0.7)	t = −1.270 *p* = 0.215
HRV (deep breathing)	28.93 (±10.8)	23.81 (±13.3)	t = 1.101 *p* = 0.281
EI	1.34 (±0.1)	1.27 (±0.1)	t = 1.176 *p* = 0.250
VI	1.73 (±0.5)	1.57 (±0.3)	t = 0.809 *p* = 0.426
HR	71 (±11.0)	68.90 (±11.2)	t = 0.480 *p* = 0.634
SBP	120 (16.5)	120 (30)	z = −0.101 *p* = 0.919
DBP	80 (20)	80 (8.7)	z = −0.167 *p* = 0.867
	Medians (IQR)		
MBP	93.33 (10.4)	93.33 (10)	z = −0.100 *p* = 0.919
SDNN	43.50 (36.7)	29.00 (25)	z = −1.308 *p* = 0.190
CV	5.05 (5.2)	3.50 (3.1)	z = −1.777 *p* = 0.075
RMSSD	31.64 (53.7)	22.63 (18.8)	z = −1.233 *p* = 0.217
Active stand HR	1.09 (0.2)	1.10 (0.4)	z = −0.123 *p* = 0.901
Active stand BP	−10.00 (13.7)	−10.00 (15.0)	z = −2.265 *p* = 0.178

Mann–Whitney T and U tests used to assess differences between groups. $\bar{x}$ = Mean, IQR = Interquartile Range, BMI = Body Mass Index, HR = Heart Rate, SBP = Systolic Blood Pressure, DBP = Diastolic Blood Pressure, MAP = Mean Arterial Pressure, SDNN = Standard Deviation of RR intervals, CV = Coefficient of Variation, RMSSD = square root of the mean value of the sum of the squared differences of the successive RR intervals, log LF = logarithm of low frequencies, log HF = logarithm of high frequencies, LF/HF = low frequencies/high frequencies, HRV (deep breathing)= Heart rate variability during deep breaths, EI = expiration/inspiration ratio, IV = Valsalva index, ΔSBP = SBP change while standing.

## Data Availability

Not applicable.

## References

[1] Bellavia S., Scala I., Luigetti M., Brunetti V., Gabrielli M., Verme L.Z.D., Servidei S., Calabresi P., Frisullo G., Della Marca G. (2021). Instrumental Evaluation of COVID-19 Related Dysautonomia in Non-Critically-Ill Patients: An Observational, Cross-Sectional Study. J. Clin. Med..

[2] Kaliyaperumal D., Rk K., Alagesan M., Ramalingam S. (2021). Characterization of cardiac autonomic function in COVID-19 using heart rate variability: A hospital based preliminary observational study. J. Basic Clin. Physiol. Pharmacol..

[3] Pellegrini L., Albecka A., Mallery D.L., Kellner M.J., Paul D., Carter A.P., James L.C., Lancaster M.A. (2020). SARS-CoV-2 Infects the Brain Choroid Plexus and Disrupts the Blood-CSF Barrier in Human Brain Organoids. Cell Stem Cell.

[4] Keyhanian K., Umeton R.P., Mohit B., Davoudi V., Hajighasemi F., Ghasemi M. (2021). SARS-CoV-2 and nervous system: From pathogenesis to clinical manifestation. J. Neuroimmunol..

[5] Lanza G.A. (2022). Autonomic dysfunction and post-COVID-19 syndrome: A still elusive link. Heart Rhythm.

[6] Dani M., Dirksen A., Taraborrelli P., Torocastro M., Panagopoulos D., Sutton R., Lim P.B. (2021). Autonomic dysfunction in ‘long COVID’: Rationale, physiology and management strategies. Clin. Med..

[7] Pan Y., Yu Z., Yuan Y., Han J., Wang Z., Chen H., Wang S., Wang Z., Hu H., Zhou L. (2021). Alteration of Autonomic Nervous System Is Associated With Severity and Outcomes in Patients with COVID-19. Front. Physiol..

[8] Becker R.C. (2021). Autonomic dysfunction in SARS-CoV-2 infection acute and long-term implications COVID-19 editor’s page series. J. Thromb. Thrombolysis.

[9] Hinduja A., Moutairou A., Calvet J.-H. (2021). Sudomotor dysfunction in patients recovered from COVID-19. Neurophysiol. Clin..

[10] Shah B., Kunal S., Bansal A., Jain J., Poundrik S., Shetty M.K., Batra V., Chaturvedi V., Yusuf J., Mukhopadhyay S. (2022). Heart rate variability as a marker of cardiovascular dysautonomia in post-COVID-19 syndrome using artificial intelligence. Indian Pacing Electrophysiol. J..

[11] Wirth K.J., Löhn M. (2022). Orthostatic Intolerance after COVID-19 Infection: Is Disturbed Microcirculation of the Vasa Vasorum of Capacitance Vessels the Primary Defect?. Medicina.

[12] Soliński M., Pawlak A., Petelczyc M., Buchner T., Aftyka J., Gil R., Król Z., Żebrowski J. (2021). Heart Rate Variability Changes in Mild-Symptomatic, Physically Fit Male in 4–6 Weeks from the End of SARS-CoV-2 Infection. https://www.researchsquare.com/article/rs-909431/v1.

[13] Kurtoğlu E., Afsin A., Aktaş İ., Aktürk E., Kutlusoy E., Çağaşar Ö. (2021). Altered cardiac autonomic function after recovery from COVID-19. Ann. Noninvasive Electrocardiol..

[14] Asarcikli L.D., Hayiroglu M.İ., Osken A., Keskin K., Kolak Z., Aksu T. (2022). Heart rate variability and cardiac autonomic functions in post-COVID period. J. Interv. Card. Electrophysiol..

[15] Townsend L., Moloney D., Finucane C., McCarthy K., Bergin C., Bannan C., Kenny R.-A. (2021). Fatigue following COVID-19 infection is not associated with autonomic dysfunction. PLoS ONE.

[16] Azabou E., Bao G., Bounab R., Heming N., Annane D. (2021). Vagus Nerve Stimulation: A Potential Adjunct Therapy for COVID-19. Front. Med..

[17] Faul F., Erdfelder E., Buchner A., Lang A.-G. (2009). Statistical Power Analyses Using G*Power 3.1: Tests for Correlation and Regression Analyses. Behav. Res. Methods.

[18] Machado García A. (2008). Optimización de los Métodos para Estudiar la Variabilidad de la Frecuencia Cardiaca y su Aplicación a Grupos de Sujetos Sanos y Enfermos [Internet]. [Cuba]: Repositorio de Tesis en Ciencias Biomédicas y de la Salud de Cuba. http://tesis.sld.cu/index.php?P=FullRecord&ID=539.

[19] Lewek J., Jatczak-Pawlik I., Maciejewski M., Jankowski P., Banach M. (2021). COVID-19 and cardiovascular complications—Preliminary results of the LATE-COVID study. Arch. Med. Sci..

[20] Bourdillon N., Yazdani S., Schmitt L., Millet G.P. (2020). Effects of COVID-19 lockdown on heart rate variability. PLoS ONE.

